# Impact of Proton Pump Inhibitor Therapy on Bone Mineral Density: An Updated Systematic Review

**DOI:** 10.7759/cureus.106698

**Published:** 2026-04-09

**Authors:** Chalini Sundar, Darshan Devang Divakar, S. Saravana Kumar, Annapoorani Manoharan, Sundar Ramalingam

**Affiliations:** 1 Dental Public Health, Faculty of Health Sciences, JSS Academy of Higher Education and Research, Vacoas-Phoenix, MUS; 2 Research and Publication Department, Saudi Dental Society, King Saud University College of Dentistry, Riyadh, SAU; 3 Oral Medicine and Radiology, Faculty of Health Sciences, JSS Academy of Higher Education and Research, Vacoas-Phoenix, MUS; 4 Pharmacology, Government Thiruvarur Medical College, The Tamil Nadu Dr. MGR Medical University, Chennai, IND; 5 Plastic and Reconstructive Surgery, Madras Medical College and Rajiv Gandhi Government General Hospital, The Tamil Nadu Dr. MGR Medical University, Chennai, IND; 6 Oral and Maxillofacial Surgery, King Saud University College of Dentistry, Riyadh, SAU; 7 Oral and Maxillofacial Surgery, Dental University Hospital, King Saud University Medical City, Riyadh, SAU

**Keywords:** bone health, bone mineral density, bone mineralization, fracture risk, osteoporosis, proton pump inhibitors

## Abstract

Proton pump inhibitors (PPIs) are often prescribed for acid-related disorders, but while chronic use may harm bone health, a clear link to changes in bone mineral density (BMD) remains unestablished. Therefore, this systematic review aims to evaluate the association between prolonged PPI therapy and changes in BMD across skeletal sites. Following PROSPERO registration (ID: CRD420250651077), a comprehensive search of PubMed/MEDLINE, Cochrane CENTRAL, Web of Science, and Scopus databases (January 2005-September 2025) identified original research studies assessing changes in BMD among adults (≥18 years) using PPIs for ≥6 months. Two reviewers independently extracted the data for qualitative data synthesis. Risk-of-bias was assessed using the ROBINS-E tool. A total of 14 studies (11 cohort, two cross-sectional, one case-control), with participants ranging from 40 to >6,000, were included. Most studies (13 out of 14; 92.9%) assessed BMD changes in the hip, femoral neck, and lumbar spine. Ten studies (71%) found significant BMD reductions among PPI users, particularly at the hip and femoral neck, and also in the mandible. Findings for lumbar spine BMD were inconsistent. Longer PPI therapy (>1 year) and higher doses were associated with greater BMD reductions. Esomeprazole and lansoprazole were linked to more significant BMD loss than omeprazole, with older adults, males, and postmenopausal women being more affected. Proposed mechanisms include impaired calcium absorption, osteoclast-mediated trabecular bone reduction, altered plasma metabolites, and inhibition of bone-specific enzymes. Prolonged PPI use ≥6 months is linked with a modest overall reduction in BMD, especially in the hip and femoral neck, which may contribute to fracture risk. Clinicians should use the lowest effective dose and duration, and regularly monitor BMD in high-risk patients using PPIs.

## Introduction and background

Proton pump inhibitors (PPIs) are among the most widely prescribed medications worldwide, primarily used for the treatment of acid-related gastrointestinal disorders [1]. PPI medications such as omeprazole, esomeprazole, lansoprazole, pantoprazole, rabeprazole, and dexlansoprazole are commonly prescribed for conditions like esophagitis, functional dyspepsia, peptic ulcer disease, gastroesophageal reflux disease (GERD), and Zollinger-Ellison syndrome [2,3]. Since the late 1980s, PPIs have largely replaced histamine-2 receptor antagonists (H2RAs) due to their superior ability to suppress gastric acid secretion and favorable short-term safety [4]. Their availability, including over-the-counter options, has led to both appropriate and sometimes unnecessary long-term use [5,6].

PPIs are prodrugs that irreversibly inhibit the H⁺/K⁺-ATPase enzyme in parietal cells, blocking gastric acid secretion and reducing acidity [2]. Because H⁺/K⁺-ATPase enzymes are present in various tissues, PPIs may also exert effects outside of the gastrointestinal system [2,7]. Prolonged use of PPIs, while generally safe, can lead to micronutrient deficiencies, as these nutrients require gastric acid for absorption [8,9]. As a result, chronic PPI therapy may lead to adverse effects, including iron, vitamin B12, and magnesium deficiencies, hypocalcemia, and increased risk of enteric and non-enteric infections [8-10]. More importantly, it could have detrimental effects on bone health, including reduced trabecular bone score (TBS), changes in bone mineral density (BMD), and increased fragility and fracture risk [2,11-13].

Concerns about skeletal health in chronic PPI users stem from gastric acidity's role in calcium absorption and subsequent bone remodeling [9,14]. By increasing gastric pH, PPIs impair calcium ionization and absorption, especially of insoluble salts, thereby lowering calcium bioavailability for bone formation [9]. Additionally, PPIs may affect bone metabolism through mechanisms such as secondary hyperparathyroidism and interference with bone-specific hormones and enzymes [15,16]. Studies have also shown changes in osteoblast and osteoclast function and in bone microarchitecture among long-term PPI users, even without notable changes in overall bone health and mineralization [7,17]. Nevertheless, the clinical significance of these biological mechanisms remains uncertain due to inconsistent findings in previous observational studies [2]. Similarly, while there is reported evidence linking prolonged PPI use to an increased risk of fractures, the strength of this association is debated because of a lack of information suggesting changes in BMD [13,18,19]. Considering the aforementioned facts, important clinical questions arise regarding the increasing long-term use of PPIs, especially among older adults and postmenopausal women who are at risk of developing osteoporosis [12].

Several previous reports have reported the adverse effects of chronic PPI use on TBS, which was used as a determinant of fracture risk based on bone microarchitecture [20-22]. However, in recent years, BMD has emerged as a critical determinant of skeletal health and is considered a benchmark diagnostic tool for the diagnosis of osteoporosis and osteopenia [20]. While both TBS and BMD can be evaluated using dual-energy X-ray absorptiometry (DEXA), BMD provides a quantitative measure (in g/cm^2^) that is comparable across longitudinal assessments and patient groups [20]. Moreover, BMD is incorporated into the FRAX tool, which is widely used to estimate long-term fracture risk [20,23]. Lastly, studies implicating chronic PPI use in increased fracture risk denied any significant association after adjusting for confounders such as age, systemic status, and BMI, leading to speculation that it could be due to a decrease in BMD [2,11,18,19]. Although individual studies and previous reviews have examined fracture risk and broader skeletal outcomes associated with PPI use, fewer have specifically focused on BMD changes across skeletal sites using updated evidence. This review was undertaken to meet the need for an updated, BMD-focused synthesis of observational evidence, with particular attention to site-specific skeletal findings and exposure characteristics.

Therefore, the present review was conducted with the hypothesis that PPI use ≥6 months contributes to compromised bone health by decreasing BMD. Accordingly, this systematic review aims to evaluate the association between prolonged PPI therapy and decreased BMD across different skeletal sites. The review further seeks to determine if variations in patient demographics, PPI drug type, dosage, duration, and measurement sites could alter the impact of prolonged PPI therapy on BMD, thereby providing insights for clinical decision-making and future research.

## Review

Methods

Focused Question

The current systematic review was designed to address the focused question, “Does prolonged use of proton pump inhibitors (PPIs) affect bone mineral density (BMD)?” Accordingly, the review identified studies that assessed the impact of PPI use (≥6 months) on BMD. Following registration of the study protocol in the International Prospective Register of Systematic Reviews (PROSPERO ID - CRD420250651077), the review was conducted in adherence to the Preferred Reporting Items for Systematic Reviews and Meta-Analyses (PRISMA 2020) checklist [24].

Search Strategy

A comprehensive search was conducted in PubMed/MEDLINE, Cochrane CENTRAL Library, Web of Science (Core collection), and Scopus (Elsevier) databases. While the initial search intentionally applied no restrictions on language, publication year, or geographic location, allowing us to capture a diverse range of studies and findings, subsequent study selection was limited to English-language articles published between January 2005 and September 2025. Although this was done due to feasibility constraints, it may have introduced language bias. Similarly, grey literature and trial registries were not systematically searched due to inconsistent evidence quality in these sources. Nevertheless, in order to ensure a more inclusive and robust dataset for our analysis, the search was conducted using the MeSH terms, including but not limited to "proton pump inhibitors," "bone mineral density," "osteoporosis," "fracture risk," and "bone mineralization”, along with different combinations of Boolean operators. The detailed initial search strategy and search strings for each database are provided in the appendices.

Studies were selected for review based on a primary eligibility criterion of being original research articles evaluating the association between chronic PPI use and BMD, and using either DEXA or any suitable investigative modality for assessing BMD, as a reference standard. Based on the PICOS framework (Population, Intervention, Comparison, Outcome, and Study setting), the inclusion criteria were as follows.

Population: Adults (≥18 years) from any clinical or community setting, who have been on PPI therapy.

Intervention: Prolonged use of PPIs (e.g., omeprazole, pantoprazole, lansoprazole, etc.), defined as daily use for ≥ 6 months. PPI use for ≥6 months was used as the minimum exposure threshold for inclusion, acknowledging variability in definitions of prolonged use across studies.

Comparison: Individuals not using PPIs or using other acid-suppressive medications (e.g., H2 receptor antagonists).

Outcomes: Changes in bone mineral density measured using dual-energy X-ray absorptiometry (DEXA) or other validated methods; Markers of bone turnover and mineralization (if available); Fracture incidence associated with PPI use (if available).

Study Design: Observational studies (cohort, case-control, and cross-sectional).

Case reports, reviews, studies lacking quantitative assessment for BMD, studies with poor methodological quality, and those without a comparison group were excluded.

Data Extraction and Quality Assessment

All retrieved records were imported into EndNote reference management software (Clarivate, London, UK) for title and abstract screening and removal of duplicates. Two independent reviewers (C.S. and S.R.) evaluated and selected the studies for inclusion, achieving a high level of inter-rater agreement with a Cohen’s kappa coefficient of 0.91. For studies deemed potentially eligible, full texts were obtained and evaluated for inclusion. Any disagreements were resolved through discussion or by consulting a third reviewer (S.S.K.). Data were independently extracted by the two reviewers (C.S. and S.R.) using a standardized data extraction form and subsequently tabulated in a spreadsheet.

The extracted data included study characteristics including author, year, study design, and sample size; population details such as age, sex distribution, baseline comorbidities (if any); exposure details (PPI type, dosage, and duration); comparator group characteristics; outcome measures like method of BMD assessment, skeletal site measured, and reported changes in BMD; and lastly the key findings and conclusions. During data extraction, no restrictions were applied based on the degree of adjustment for confounding variables to capture the full scope of available evidence; however, this was considered during the interpretation of findings.

Risk of Bias Assessment

The potential for bias in the non-randomized studies of exposure was evaluated using the Cochrane ROBINS-E tool, which identifies risks across seven key areas: (i) confounding factors, (ii) exposure measurement, (iii) selection of participants, (iv) post-exposure interventions, (v) incomplete data, (vi) measurement of outcomes, and (vii) selection of reported results [25].

Each area was categorized as having low risk, some concerns, high risk, or very high risk of bias, following the guidance of the ROBINS-E tool. An overall risk assessment for each study was then determined by selecting the highest level of risk across the different areas. The two reviewers mentioned earlier conducted independent assessments of each study to identify the risk of bias across the seven domains of the ROBINS-E tool, and any disagreements were resolved through discussion and consensus. The findings from the risk-of-bias evaluation were illustrated with visual plots to provide both domain-specific and overall study-level insights, using the “robvis” web-based visualization tool [26].

Assessment of Heterogeneity and Data Synthesis

Given the inclusion of observational studies with varying designs (cohort, cross-sectional, and case-control), substantial methodological and clinical heterogeneity was anticipated. Sources of heterogeneity included differences in study populations (age, sex distribution, comorbidities), duration and dosage of PPI exposure, and methods and anatomical sites for BMD measurement. Due to this variability, a quantitative meta-analysis was not performed. Instead, a qualitative synthesis approach was adopted. Findings were interpreted by grouping studies according to key characteristics, including study design, exposure duration, and anatomical site of BMD assessment. Greater emphasis was placed on identifying patterns of consistency or inconsistency across studies rather than deriving pooled effect estimates.

Since the available evidence base was limited, all eligible observational studies were included irrespective of the degree of covariate adjustment. However, differences in adjustment for age, BMI, comorbidities, and indication for PPI use were considered during narrative interpretation, as these factors may introduce residual confounding. Furthermore, due to variability in reporting of exposure duration across studies, stratified analysis based on duration (e.g., ≥1 year or ≥5 years) was not feasible. Additionally, variability in BMD assessment methods and skeletal sites, and variations in outcome measurement techniques (e.g., DEXA vs. 3D-QCT vs. radiographic indices) and differences in comparator groups were considered when qualitatively interpreting the strength and direction of associations.

Results

Study Selection

**Figure 1:**
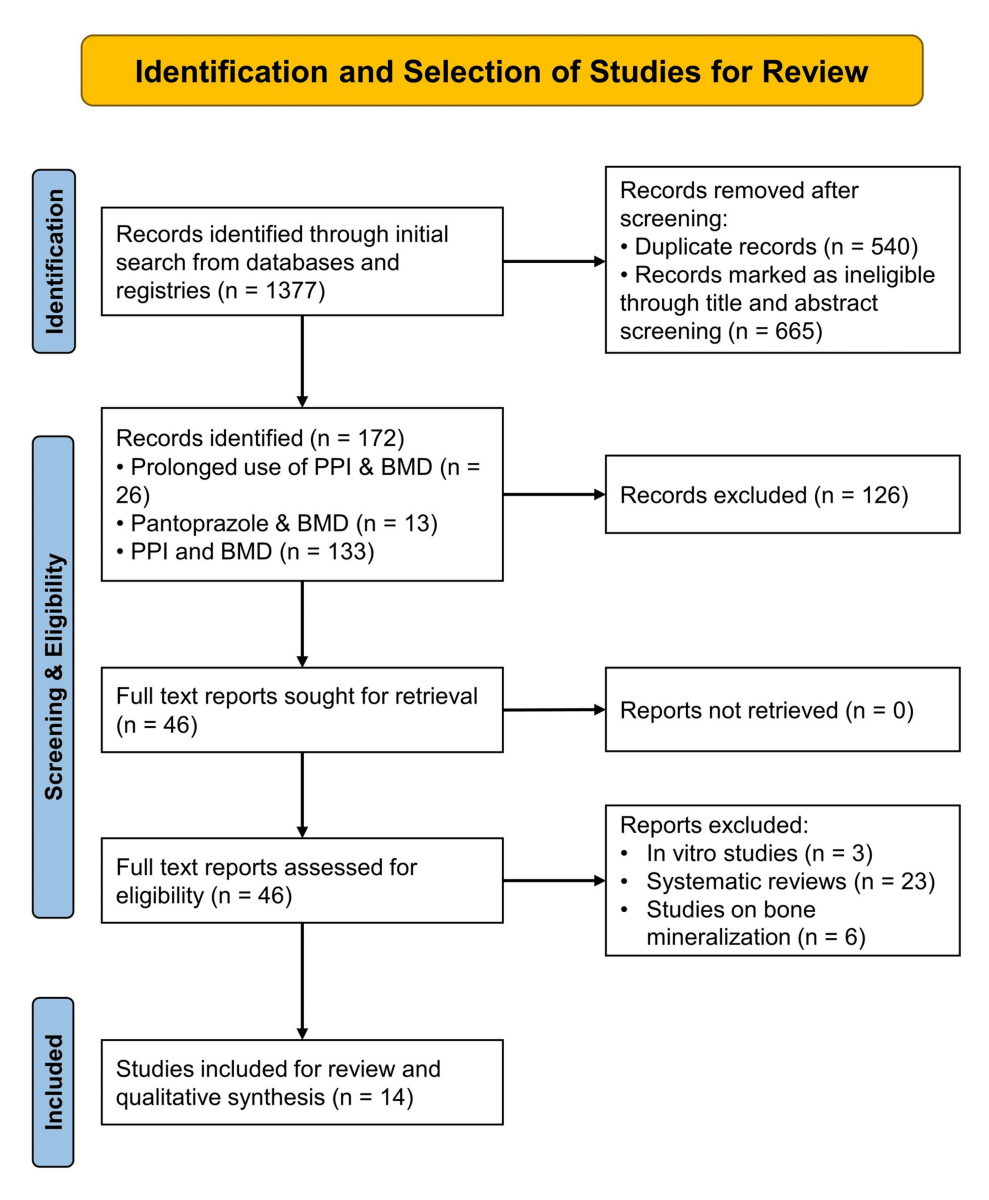
PRISMA flowchart depicting screening, identification, exclusion and selection of the studies for systematic review. PPI, proton pump inhibitor(s); BMD, bone mineral density

Based on an initial electronic search across multiple databases, 1377 records were identified; 1205 were excluded during title and abstract screening (n=665) and deduplication (n=540). A further 126 records were excluded for not fulfilling the inclusion criteria, and 46 potential articles were selected for retrieval. Among these, 32 studies were excluded due to the removal of in vitro studies (n=3), reviews (n=26), and studies on bone mineralization but not related to PPI use (n=6). Finally, data from 14 eligible studies were included for systematic review, data extraction, and qualitative synthesis. The PRISMA flow chart depicting study screening, identification, exclusion, and selection is shown in Figure 1.

Overall Study Characteristics

Among the 14 selected studies that fulfilled the inclusion criteria, 11 were cohort studies (both prospective and retrospective) [4,21,27-35], two were cross-sectional studies [36,37], and one was a case-control study [14], all published between 2008 and 2025. While the sample sizes of these studies in the PPI user groups ranged from 40 to more than 3,000 patients, the non-PPI user sample sizes were mostly unmatched and ranged from 40 to over 100,000 patients. The study samples included participants of both sexes, with mean ages varying from 20 to 70 years. The duration of PPI exposure ranged from 8 months to over 10 years. The commonly evaluated medications included omeprazole, esomeprazole, lansoprazole, pantoprazole, rabeprazole, and dexlansoprazole. Bone mineral density was predominantly assessed using DEXA, although one study used 3D quantitative computed tomography (3D-QCT) [33], and another used dental panoramic radiography to assess mandibular bone [37]. The aforementioned measurement differences contributed to methodological heterogeneity and limited direct comparability across studies. Table 1 and Table 2 summarize and describe the extracted data from the included studies.

**Table 1:** Characteristics of the included studies, including study design, sample demographics, and proton-pump inhibitor usage. PPI: proton pump inhibitor(s); H2RA: histamine-2 receptor antagonist

Study (Author, Year)	Study Design	Sample Size (n)		Age of Study Population	PPI Type and Duration
		PPI users	Non-users		
Yu et al. (2008) [27]	Prospective cohort	Females: 234; males: 487	Females: 4,574; males: 4,920	Age ≥ 65 years	Type, dose and strength of PPI used were not mentioned.
Gray et al. (2010) [28]	Prospective cohort	3,396	148,394	Mean age: PPI users, 64.8 ± 7.1 years; PPI non-users - 63.1 ± 7.3 years	Omeprazole, esomeprazole, lansoprazole, pantoprazole, rabeprazole. The duration ranged from less than one year, 1-3 years and more than 3 years.
Targownik et al. (2012) [29]	Prospective cohort	228	4,284	Age ≥ 25 years	Type, dose and strength of PPI used were not mentioned.
Ozdil et al. (2013) [30]	Prospective cohort	114	110	Mean age: 37.7 ± 8.8 years	Lansoprazole 30 mg/day, pantoprazole 40 mg/day, or esomeprazole 40 mg/day. Mean duration was 8.5 ± 2.3 months.
Solomon et al. (2015) [31]	Prospective cohort	207	1,676	Mean age: PPI users, 50.7 ± 4.2 years; PPI non-users, 50.2 ± 3.9 years	Comparison between PPI and H2RA. Type, dose and strength of PPI or H2RA used were not mentioned. Study duration was 9.9 years.
Arj et al. (2016) [36]	Cross-sectional	40	40	Age range: 20 to 45 years	Type, dose and strength of PPI used were not mentioned. Study duration was ≥ 2 years.
Bahtiri et al. (2016) [32]	Prospective cohort	200	50	Mean age: 50.6 ± 10.6 years	Omeprazole, esomeprazole, lansoprazole, and pantoprazole, for a duration of 12 months.
Targownik et al. (2017) [33]	Prospective cohort	52	52	Mean age: PPI users, 65.1 ± 9.1; PPI non-users, 64.9 ± 7.9	Type, dose and strength of PPI used were not mentioned. Study duration was 5 years.
Shin et al. (2019) [21]	Retrospective cohort	223 (All females)	223 (All females)	Mean age: 64.3 ± 10.4 years	Type, dose and strength of PPI used were not mentioned. Study duration was one year.
Coşgunarslan et al. (2021) [37]	Cross-sectional	201	201	Median age: 42.5 years	Esomeprazole (40.8%), lansoprazole (28.9%), pantoprazole (21.4%), rabeprazole (8%) or omeprazole (1%), for a duration of 12 months.
Gao et al. (2021) [4]	Retrospective cohort	381 (All males)	3,682 (All males)	Age ≥ 70 years	Omeprazole, esomeprazole, lansoprazole, pantoprazole, and rabeprazole, for a duration of 12 months.
Zhang et al. (2023) [34]	Retrospective cohort	1,292	6,446	Age range: 49.7 to 62.5 years	Type, dose and strength of PPI used were not mentioned. Study duration was 3.5 years.
Smaoui et al. (2024) [14]	Case-control study	90	90	Mean age: 55 ± 12.22 years	Patients treated with PPIs for ≥1 year, including omeprazole 81.1% and esomeprazole 18.9%.
Bioletto et al. (2025) [35]	Retrospective cohort	Females: 232; males: 252	6,994	Age range: 44.3 to 58.3 years	Omeprazole (n = 161), followed by esomeprazole (n = 141), lansoprazole (n = 83), pantoprazole (n = 59), and rabeprazole (n = 40), for a duration of 12 months.

**Table 2:** Characteristics of the included studies, including bone mineral density measurements, study outcomes, and key findings. PPI, proton pump inhibitor(s); BMD, bone mineral density; DEXA, dual-energy x-ray absorptiometry; H2RA, histamine-2 receptor antagonist; 3D-QCT, quantitative computed tomography; ROI, region of interest; MCW, mandibular cortical width; KI, Klemetti index

Study (Author, Year)	BMD Measurement		Outcomes	Key Findings
	Modality	Sites		
Yu et al. (2008) [27]	DEXA	Total hip and femoral neck	Significant decrease in BMD among men (0.946; p<0.05) when compared to women (0.729; p=0.37).	PPIs are associated with a modest decrease in bone density in men, while no similar effects have been observed in women. Additionally, the use of acid-suppressive medications is linked to a slight increase in non-spine fracture risk, which is observed in both men and women with low calcium intake.
Gray et al. (2010) [28]	DEXA	Hip, spine, forearm, and wrist	BMD measurements were similar between PPI users and nonusers at baseline. PPI use had a marginal effect on 3-year BMD change at the hip (p=0.05) but not at other sites, and this effect was not observed at the 6-year follow-up.	Prolonged PPI use does not demonstrate a significant association with the occurrence of hip fractures; however, there is a modest clinical association between prolonged PPI use and the incidence of fractures involving the spine, forearm, and wrist.
Targownik et al. (2012) [29]	DEXA	Femoral neck, total hip, and lumbar spine (L1-L4)	PPI use significantly decreased BMD at the hip (0.019±0.008 g/cm²; p<0.05) and femoral neck (0.022±0.007 g/cm²; p<0.01), but not at the lumbar spine (0.014±0.011 g/cm²; p=0.179).	Prolonged use of PPIs over a 10-year period was associated with a modest yet statistically significant reduction in BMD, with this decrease observed specifically in the hip and femoral neck regions, while no significant changes were noted in the lumbar spine; additionally, restricting the analysis to subjects aged 50 years and older did not have a considerable impact on the parameter estimates related to PPI use.
Ozdil et al. (2013) [30]	DEXA	Lumbar spine and femoral neck	Significant reduction in BMD was observed in the vertebral and femoral bones among PPI users. Median reduction in T-score (vertebra, 0.23 ± 0.42 units; 95% CI 0.15-0.30; p<0.01 / femur, 0.10 ± 0.40 units; 95 % CI 0.03-0.18; p<0.05).	A significant overall reduction in BMD was observed in both the vertebrae and femur; T-score analysis indicated that the reduction in BMD following treatment with lansoprazole was significantly greater than that observed with pantoprazole, particularly in the lumbar spine and vertebrae, while in the femur, the reduction in BMD was greater in the esomeprazole group compared to the lansoprazole and pantoprazole groups, although this difference did not reach statistical significance.
Solomon et al. (2015) [31]	DEXA	Lumbar spine, femoral neck, or total hip	No significant decrease in BMD was observed in the lumbar spine, femoral neck, and total hip regions among users of acid-suppressive medications compared to non-users. There was also no significant difference in BMD changes between PPI and H2RA users.	The annualized change in BMD at the lumbar spine, femoral neck, and total hip regions among users of PPIs is similar to that observed in both H2RA users and non-users, indicating that the effects of PPIs and H2RAs on BMD are comparable, with no significant impact on bone health based on the type of acid-suppressing medication used.
Arj et al. (2016) [36]	DEXA	Femur and lumbar spine	Femoral BMD showed a statistically significant decrease among PPI users, while it increased in non-users during the same period. Mean change in T-score (PPI users -0.44 ± 1.11 / non-users +0.19 ± 0.95; p<0.01). No significant difference in lumbar spine BMD between PPI users and non-users.	Prolonged use of PPIs was associated with a significant reduction in femoral BMD compared to non-users, while a decrease in lumbar spine BMD was also observed, although this difference did not reach statistical significance relative to non-users.
Bahtiri et al. (2016) [32]	DEXA	Femoral neck and total hip	Significant reduction in BMD was observed in both the femoral neck and total hip regions. Mean reduction in T-score (Femoral neck - 2.764; p<0.01 / Total hip - 3.281; p<0.01).	The overall analysis indicates a significant reduction in BMD across the femoral neck and total hip regions, with variability in BMD reduction observed among different PPI medications; esomeprazole was independently associated with a notable decline in BMD, whereas omeprazole did not demonstrate any significant effects on BMD.
Targownik et al. (2017) [33]	DEXA and volumetric BMD using 3D-QCT	Femoral neck and total hip	PPI use was neither associated with a significant decrease in mean BMD nor with a significantly increased risk of fractures.	Prolonged use of PPIs for periods of 5 and 10 years did not result in a significant acceleration in covariate-adjusted BMD loss, and there was no evidence of an increased risk for fractures involving the femoral neck and total hip regions.
Shin et al. (2019) [21]	DEXA	Total hip, femoral neck and trochanter, and lumbar spine (L1 - L4)	Based on T-score, a decrease in BMD was observed among PPI users in the total hip (0.32 ± 1.01), femoral trochanter (0.20 ± 0.97), and lumbar spine (0.73 ± 1.30); and femoral neck BMD increased marginally (0.13 ± 0.89). The difference in BMD between PPI users and non-users was statistically significant only for the total hip.	Prolonged PPI use resulted in a decrease in BMD in the total hip, femoral trochanter, and lumbar spine regions, while femoral neck BMD showed only a marginal increase; total hip BMD was significantly lower in the PPI exposure groups regardless of exposure timing, although the association between BMD and PPI exposure timing was inconsistent for the lumbar spine and femoral neck.
Coşgunarslan et al. (2021) [37]	Panoramic X-rays	Four ROI in the mandible: (1) upper part of ramus; (2) angle; (3) anterior rim of mental foramen; and (4) distal border of middle ramus.	Significant differences were observed in terms of ROI3, MCW, and KI between the control and study groups (p < 0.05). However, there were no significant differences found for ROI1, ROI2, ROI4, and PMI (p > 0.05). Additionally, males were more severely affected than females.	Trabecular and cortical bones in the mental foramen region showed significant osteoporotic changes in PPI users, while no notable differences in bone density or structure were observed in the mandibular ramus and angle regions, highlighting concerns regarding the impact of PPIs on jawbone health.
Gao et al. (2021) [4]	DEXA	Lumbar spine	Long-term use of PPIs is associated with lower BMD in the lumbar spine. Analysis of the association between PPI and BMD showed that the estimates remained statistically significant in males (-0.0604 (-0.0855, -0.0354)), individuals aged over 70 years (-0.0390 (-0.0643, -0.0137)), and White participants (-0.0225 (-0.0437, −0.0012)).	Prolonged use of PPIs is associated with lower lumbar spine BMD, particularly in men and individuals over 70 years of age, whereas H2RAs do not significantly affect lumbar spine BMD; these findings have clinical implications, suggesting that PPI use should be limited to less than one year in high-risk patients, with consideration given to switching to H2RAs when feasible.
Zhang et al. (2023) [34]	DEXA	Lumbar spine, femoral neck, and total hip	There was no difference in lumbar spine BMD between PPI users and non-users. However, a significant decrease in BMD was observed among PPI users in the femoral neck (0.009 ± 0.004; p<0.05) and total hip (0.012 ± 0.004; p<0.01). PPI induced changes in plasma metabolite levels of nine lipids associated with total hip BMD.	A notable decrease in lumbar spine and total hip BMD was observed among PPI users, with PPI use affecting total hip BMD both directly and indirectly, primarily through the regulation of plasma metabolites, particularly those involved in the sex hormone (DHAS, dehydroisoandrosterone sulfate) pathway.
Smaoui et al. (2024) [14]	DEXA	Lumbar spine and femoral neck	Multivariate regression analysis, adjusted for demographic factors, physical and systemic status, menopause, type of PPI, treatment modality, and vitamin D levels, showed a significant decrease in BMD among PPI users aged over 50 years (p<0.05).	There is a risk of decreased bone mineral density (BMD) in patients undergoing prolonged PPI treatment, with this risk further exacerbated by factors including age over 50 years, menopause, limited sun exposure, higher PPI doses, daily PPI intake, post-meal PPI use, and concomitant use of PPIs with a mucoprotective agent.
Bioletto et al. (2025) [35]	DEXA	Lumbar spine and femoral neck	A statistically significant association was found between chronic use of PPIs and a decrease in trabecular bone score. This association remained significant even after further adjusting for BMD at both the lumbar spine and femoral neck (-0.026; 95% CI, -0.039 to -0.012; P = 0.001), but it was observed only in the male sample.	Chronic use of PPIs affects trabecular bone quality in men independent of BMD, with impairment of bone quality being more pronounced than bone mass loss, while no association has been found between PPI use and trabecular bone quality or BMD in females, regardless of menopausal status.

Risk of Bias Assessment

**Figure 2:**
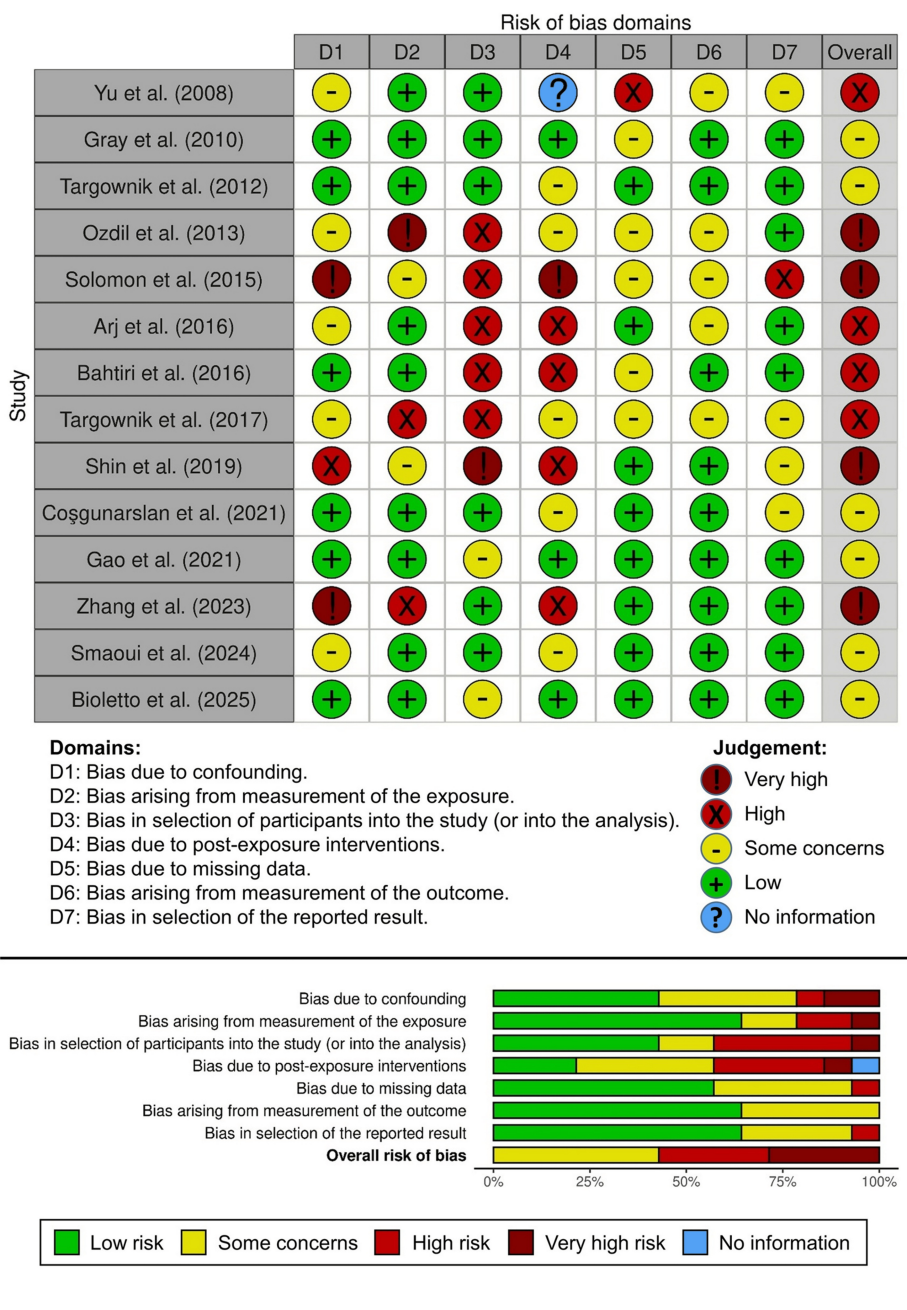
Risk-of-bias assessment using the Cochrane ROBINS-E tool for included observational studies showing domain-specific judgments for each study (top) and summary of the overall proportion of studies with low, moderate, or high risk across all bias domains (bottom).

The risk-of-bias assessment using the ROBINS-E tool, summarized in Figure 2, revealed considerable heterogeneity in methodological quality among the included observational studies. The included studies were judged to have either some concerns (6 studies, 42.8%), a high risk of bias (4 studies, 28.6%), or a very high risk of bias (4 studies, 28.6%), despite a few studies achieving low-risk-of-bias ratings in some domains. Based on some concerns and a high or very high risk of bias, the most frequent sources of bias were post-exposure interventions (D4; 11 studies), followed by participant selection (D3; 8 studies) and confounding (D1; 8 studies). On the contrary, bias arising from measurement of exposure (D2; 9 studies), measurement of outcomes (D6; 9 studies), selection of reported results (D7; 9 studies), and missing data (D5; 8 studies) showed only a low risk in the majority of the included studies. Nevertheless, the overall risk indicated a predominantly high or very high risk of bias, highlighting that while several studies were robust, more than half of the included studies exhibited significant biases that may undermine the reliability of the pooled conclusions.

*Relationship Between Prolonged* *PPI Use and BMD*

Although research findings on the relationship between prolonged PPI use and BMD are mixed, they suggest a modest inverse association that should be interpreted in light of variations across study designs, populations, and measurement approaches. Of the 14 included studies, 10 (71%) reported a statistically significant reduction in BMD among PPI users [4,14,21,27,29,30,32,35-37], while 4 (29%) did not report a significant association [28,31,33,34]. Several studies reported on age and sex-related vulnerabilities to decreased BMD among PPI users. Studies by Yu et al. (2008) and Bioletto et al. (2025) indicated a significant reduction in BMD among male PPI users, in comparison to females, and this was in addition to an overall decrease in trabecular bone quality [27,35]. Similarly, age was identified as a crucial factor influencing the decline in BMD following chronic PPI therapy. Studies by Gao et al. (2021) and Smaoui et al. (2024) reported that PPI users aged 50 years or older, particularly postmenopausal women, experienced a greater decrease in BMD (Table 3) [4,14].

Regarding anatomic site-specific effects, most studies reported BMD changes in either the hip (femoral neck and trochanter) or the lumbar spine. The only exceptions were the studies by Gray et al. (2010) [28], who evaluated the forearm and wrist in addition to the hip, and Coşgunarslan et al. (2021) [37], who evaluated the mandible exclusively. Among these studies, most have demonstrated significant reductions in BMD at the hip and/or femoral neck, among prolonged PPI users [21,27,29,32,34,36]. Targownik et al. (2012) and Zhang et al. (2023) observed mean decreases in BMD of 0.019-0.022 g/cm² and 0.009-0.012 g/cm², respectively, confirming that the hip was the most affected region in response to chronic PPI therapy [29,34]. However, the findings regarding lumbar spine BMD were less consistent. While some studies found statistically significant BMD loss in the lumbar spine, particularly among older adults and men [4,14,30], others found no meaningful difference in BMD between PPI users and non-users [31,34]. Other than the hip, femur, and lumbar spine, a small but non-significant decrease in BMD was reported in the wrist, and significant deterioration in mandibular BMD was observed in the mental foramen region [28,37].

Effect of PPI Type, Dose, and Duration on BMD

Only a few of the reviewed studies provided comprehensive PPI dosage data. Among them, research by Ozdil et al. (2013) and Bahtiri et al. (2016) suggested that esomeprazole and lansoprazole were associated with greater reductions in BMD compared to omeprazole or pantoprazole [30,32]. PPI use lasting 1 year or more was consistently associated with significant changes in BMD. On the contrary, shorter courses lasting less than 12 months had minimal impact [30]. In addition, two studies compared prolonged PPI use with that of H2-receptor antagonists (H2RAs) [4,31]. Both studies reported that H2RAs had similar or less pronounced effects on BMD, suggesting a potential class-specific mechanism related to extended periods of acid suppression (Table 3).

**Table 3:** Summary of findings based on the reviewed studies.

Review parameter	Findings
Number of studies reporting decreased BMD after PPI use	10/14 (71%)
Number of studies reporting no significant effect	4/14 (29%)
Regions predominantly affected among the evaluated anatomic sites	Femoral neck and total hip
Mean BMD reduction (hip/femoral neck)	0.009-0.022 g/cm² over 1-10 years
Average T-score change (when reported)	-0.10 to −0.44 units
Demographic groups most commonly affected	Patients aged > 50 years, especially postmenopausal women and elderly men
PPIs most associated with lower BMD	Esomeprazole, lansoprazole
Duration threshold for effect	≥ 1 year of continuous use
Sites with consistent null results	Lumbar spine (variable), wrist, forearm
Fracture risk findings	Only two studies reported on fracture risk. They found no increased risk for fractures despite decreased BMD [28,33].

Mechanistic Insights

Only two of the reviewed studies reported novel insights into the possible mechanisms underlying the deleterious effects of prolonged PPI use on BMD. Zhang et al. (2023) identified alterations in plasma lipid metabolites linked to the sex hormone (DHAS, dehydroisoandrosterone sulfate) pathway, suggesting that these changes may modulate bone metabolism and mineralization [34]. Additionally, Bioletto et al. (2025) demonstrated a decreased TBS value independent of BMD, indicating an overall compromise in bone microarchitecture leading to mineral loss [35].

Discussion

Since their introduction, almost four decades ago, PPIs have become widely utilized for the treatment of gastrointestinal acid-related conditions, including GERD, erosive gastritis, esophagitis, and peptic ulcer disease [1]. These medications are prescribed worldwide and available over the counter (OTC) in many regions due to their efficacy and well-established safety profiles [5,8,38]. However, over the last two decades, studies have reported several adverse effects associated with prolonged PPI use [8]. These include an elevated fracture risk attributable to PPI-induced deterioration of skeletal health, deficiencies of calcium, iron, vitamin B12, and magnesium, and even pneumonia [9,10,39]. The findings of this systematic review indicate that prolonged PPI use is associated with a modest reduction in BMD, particularly at the hip and femoral neck. Approximately 71% of studies reported a significant decline in BMD, with a cumulative impact that may become clinically significant despite a small overall reduction [4,14,21,27,29,30,32,35-37]. Although findings for lumbar spine BMD were inconclusive [31,34], a few studies reported a decline in trabecular bone quality, particularly in older men and postmenopausal women receiving PPI therapy for extended periods of time [4,14,30]. This not only reflects likely anatomic differences among bones in their response to chronic PPI exposure, but could also be related to the duration of exposure, wherein BMD loss may not have been discernible among short-term PPI users, but could be profound in the long term. Additionally, bone quality may deteriorate before any significant loss in BMD, suggesting caution for long-term and high-dose PPI users [35,40]. All of this makes it alluring to hypothesize that an impaired mineralization secondary to chronic PPI therapy might contribute to clinically increased fragility, especially in older adults and postmenopausal women [2,35,41].

In addition to BMD, TBS has also been reported as a quantitative measure of fracture risk in patients with skeletal illnesses and those at risk of developing osteoporosis [20]. However, compared with BMD, TBS is not considered an indicator of overall bone quality, as reductions in TBS have been reported without an apparent reduction in BMD [23,35,42]. Notably, Gray et al. (2010) and Targownik et al. (2017) found no significant increase in fracture risk despite reduced BMD, suggesting that PPIs may not affect bone quality beyond mineral density [28,33]. On the contrary, Bioletto et al. (2025) indicated structural deterioration in chronic male PPI users through reduced trabecular bone score [35]. Similar findings were reported by Coşgunarslan et al. (2021), who demonstrated thinning of the mandibular trabecular bone, underscoring the broader skeletal effects of chronic PPI use [37]. These alternating findings regarding reductions in TBS and BMD in prolonged PPI users, with or without correlation, indicate a greater role for PPIs in adversely affecting bone physiology and warrant further investigation [21].

Multiple systematic reviews and observational studies have linked prolonged PPI use to an increased risk of osteoporotic fractures [10,12,19,39,43]. Long-term use is associated with lower serum calcium and vitamin B12 levels, impaired collagen deposition, and reduced bone strength [9,13]. Emerging evidence indicates that PPIs may modulate gene expression, leading to decreased levels of bone formation markers [44]. Notably, hyperparathyroidism-like bone changes have been observed after more than six months of PPI use, affecting both young and older adults [15,45]. Aleraij et al. (2020) reported significant reductions in BMD among long-term PPI users, with little impact noted for those with less than six months of use [19]. Fattahi et al. found diminished bone mineral content, resembling chronic osteoporosis, particularly in the femoral neck, underscoring caution regarding PPI overuse [46]. Conversely, Paudel et al. (2023) and Kondapalli et al. (2023) found no significant associations between PPI use and bone health or microarchitecture, suggesting the need for BMD assessments in PPI users prior to orthopedic interventions [40,43]. These conflicting findings highlight the importance of the current review's results and also underscore the justification for identifying PPI use greater than six months as a criterion for prolonged PPI use.

The association between PPI use and bone effects is primarily linked to reduced gastric acidity, which decreases ionized calcium absorption and affects bone remodeling [2,18,47]. Chronic PPI therapy may lead to secondary hyperparathyroidism, increased bone turnover, and resorption [8,15,21,48,49]. Inhibition of osteoclastic vacuolar H⁺/K⁺-ATPase can impair bone resorption, while impacts on PHOSPHO1 and osteoblastic mineralization disrupt bone formation [9,17,18,50]. Additionally, age and sex-dependent effects on BMD have been observed among elderly male and postmenopausal female PPI users, indicating hormonal influences [4,14,27,35]. Zhang et al. (2023) noted that lipid metabolite changes induced by PPIs could affect DHAS pathways and BMD [34]. Ashfaq et al. (2023) found significant variations in serum sex hormone-binding globulin and testosterone levels among long-term PPI users, suggesting potential endocrine disruption in males [16]. Specific medications such as esomeprazole and lansoprazole were associated with BMD reductions, whereas omeprazole showed neutral effects [30,32]. These differences may significantly influence patients on long-term therapy, contributing to BMD changes and increased fracture risk [2,12,18]. Moreover, PPIs may affect gene expression related to collagen synthesis and extracellular matrix organization, leading to microarchitectural compromise without significant BMD deterioration [2,7,17,44,47].

Clinical Implications

While PPIs are essential for managing acid-related disorders, clinicians should carefully evaluate the benefits against potential risks to skeletal health, particularly in high-risk populations [2]. Given the widespread and often unnecessary chronic use of PPIs, the findings of the present review have significant clinical implications. Prescribers should evaluate the ongoing need for PPI therapy, particularly in patients with asymptomatic or mild gastroesophageal reflux disease, and consider implementing step-down or on-demand regimens. Additionally, it is essential to screen for BMD loss or trabecular bone changes similar to hyperparathyroidism in at-risk populations of PPI users, such as postmenopausal women, older men, and chronic corticosteroid users [9,15,51]. Furthermore, encouraging adequate intake of calcium and vitamin D, along with lifestyle modifications such as weight-bearing exercise and smoking cessation, is crucial. Lastly, prescribers might consider alternative acid-suppressive agents, such as H2RAs, which appear to have a lesser impact on BMD than PPIs, when indicated for long-term use [4,31].

Limitations and Future Directions

The heterogeneity among studies in this review is a major limitation, attributable to differences in study designs, demographics, follow-up durations, and control for confounders such as diet and nutritional status. The absence of pooled estimates further limits statistical quantification of effect size and formal assessment of between-study heterogeneity. However, cohort studies with follow-up periods of ≥5 years (e.g., Targownik et al. 2012; 2017) [29,33] reported smaller BMD changes than shorter studies (e.g., Ozdil et al. 2013; Arj et al. 2016) [30,36]. This suggests that BMD reduction may plateau at a stable exposure level. Similarly, methodological differences in DEXA measurement sites and inconsistent reporting of PPI dosage and duration further complicate comparisons. Nevertheless, collective evidence suggests that long-term PPI use is associated with a modest (≈1-3%) reduction in BMD and a significant 30-40% increase in fracture risk in older adults [12,13,39,43]. Notably, BMD loss alone does not fully account for fracture risk, as changes in bone geometry, turnover rate, and trabecular integrity are also significant factors [2,12,13,18,39,43].

Most studies included in this review were observational, introducing a considerable risk of bias. The main source of confounding was inadequate adjustment for baseline bone health, comorbidities, diet, physical activity, medications affecting bone metabolism, and indication for PPI use. Selection bias and post-exposure interventions were also frequent due to poorly defined inclusion criteria and limited control of subsequent treatments influencing BMD. Conversely, risks from missing data and outcome measurement were generally low, likely owing to standardized DEXA-based assessments. Exposure measurement bias varied, with lower risk in studies using prescription databases than those relying on self-reported PPI use. Selective reporting was mostly low to moderate. 

These findings highlight the methodological challenges of observational research on long-term PPI exposure and bone health, and also reduce confidence in causal interpretation. Heterogeneity in PPI types, doses, durations, and outcome measures further limits quantitative synthesis. Therefore, future research should prioritize standardized exposure definitions, long-term longitudinal designs (>10 years), and comparative analyses across PPI classes. Incorporation of advanced bone metrics (e.g., bone turnover markers, TBS, HR-pQCT) and molecular biomarkers (e.g., PHOSPHO1, collagen peptides) is recommended, alongside well-powered randomized controlled trials focusing on BMD and fracture outcomes.

## Conclusions

In conclusion, the findings of this review suggest a modest overall trend towards reduced BMD in prolonged PPI users, particularly in the hip and femoral neck. Nevertheless, these findings should account for heterogeneity and inconsistency across the included studies. Although the overall decrease in BMD is small, its clinical significance is particularly concerning for older adults, postmenopausal women, and chronic PPI users. The mechanisms behind these changes are multifaceted, involving both impaired calcium absorption and direct inhibitory effects on enzymes responsible for bone formation and resorption. Therefore, clinicians should closely monitor the bone health of patients who are on PPI therapy for extended periods of time, use the lowest effective dose for the shortest necessary duration, and consider preventive strategies to reduce the risk of skeletal complications.

## Appendices

Table 4 shows the initial search strategy employed in four databases.

**Table 4:** Initial search strategy in the different databases and their results.

Database	Final Search String	Date	Search Results
PubMed / MEDLINE	("Proton Pump Inhibitors"[Mesh] OR "proton pump inhibitor"[tiab] OR omeprazole[tiab] OR esomeprazole[tiab] OR lansoprazole[tiab] OR pantoprazole[tiab] OR rabeprazole[tiab] OR dexlansoprazole[tiab] OR "acid suppress*"[tiab]) AND ("Bone Density"[Mesh] OR "bone mineral density"[tiab] OR "bone mineral content"[tiab] OR BMD[tiab] OR "bone mass"[tiab] OR densitometry[tiab] OR "dual energy X-ray absorptiometry"[tiab] OR DXA[tiab] OR DEXA[tiab] OR osteopenia[tiab] OR osteoporosis[tiab]) AND (long-term[tiab] OR "long term"[tiab] OR chronic[tiab] OR prolonged[tiab] OR maintenance[tiab] OR "long-term use"[tiab] OR duration[tiab])	30 September 2025	159
Cochrane Library (CENTRAL)	("proton pump inhibitor" OR omeprazole OR esomeprazole OR lansoprazole OR pantoprazole OR rabeprazole OR dexlansoprazole) AND ("bone mineral density" OR "bone mineral content" OR BMD OR "bone mass" OR densitometry OR "dual energy x-ray absorptiometry" OR DXA OR DEXA OR osteopenia OR osteoporosis) AND (long-term OR "long term" OR chronic OR prolonged OR maintenance OR "long-term use" OR duration)	30 September 2025	18
Web of Science Core Collection	TS=(("proton pump inhibitor" OR omeprazole OR esomeprazole OR lansoprazole OR pantoprazole OR rabeprazole OR dexlansoprazole) AND ("bone mineral density" OR "bone mineral content" OR BMD OR "bone mass" OR densitometry OR "dual energy x-ray absorptiometry" OR DXA OR DEXA OR osteopenia OR osteoporosis) AND (long-term OR "long term" OR chronic OR prolonged OR maintenance OR "long-term use" OR duration))	30 September 2025	195
Scopus (Elsevier)	TITLE-ABS-KEY(("proton pump inhibitor" OR omeprazole OR esomeprazole OR lansoprazole OR pantoprazole OR rabeprazole OR dexlansoprazole) AND ("bone mineral density" OR "bone mineral content" OR BMD OR "bone mass" OR densitometry OR "dual energy x-ray absorptiometry" OR DXA OR DEXA OR osteopenia OR osteoporosis) AND (long-term OR "long term" OR chronic OR prolonged OR maintenance OR "long-term use" OR duration))	30 September 2025	1005
